# Effects of 9*cis*,11*trans *and 10*trans*,12*cis *CLA on osteoclast formation and activity from human CD14+ monocytes

**DOI:** 10.1186/1476-511X-8-15

**Published:** 2009-04-29

**Authors:** Ilana Platt, Ahmed El-Sohemy

**Affiliations:** 1 Department of Nutritional Sciences, University of Toronto, 150 College Street, Toronto, Ontario M5S 3E2 Canada

## Abstract

**Background:**

Mixed CLA isomers variably affect bone resorption in animals and decrease osteoclast formation and activity in murine osteoclasts. These variable effects may be due to the different isomers present in commercial preparations of CLA, and the effects of the predominant individual isomers, 9*cis*,11*trans *(9,11) and 10*trans*,12*cis *(10,12) CLA are not clear. The objectives of this study were to determine the effects of the individual CLA isomers on osteoclast formation and activity from human CD14^+ ^monocytes, and to determine whether any changes are accompanied by changes in cathepsin K, matrix metalloproteinase-9 (MMP-9), receptor activator of NF-κB (RANK) and tumour necrosis factor alpha (TNFα) gene expression. Osteoclasts were identified as TRAP^+ ^multinucleated cells. Osteoclast activity was quantified by the amount of TRAP in the cultured media.

**Results:**

At 50 μM, 9,11 CLA inhibited osteoclast formation by ~70%, and both 9,11 and 10,12 CLA decreased osteoclast activity by ~85–90%. Both isomers inhibited cathepsin K (50 μM 9,11 by ~60%; 10,12 by ~50%) and RANK (50 μM 9,11 by ~85%; 50 μM 10,12 by ~65%) expression, but had no effect on MMP-9 or TNFα expression.

**Conclusion:**

9,11 CLA inhibits osteoclast formation and activity from human cells, suggesting that this isomer may prevent bone resorption in humans. Although 10,12 CLA did not significantly reduce osteoclast formation, it reduced osteoclast activity and cathepsin K and RANK expression, suggesting that this isomer may also affect bone resorption.

## Background

Dietary conjugated linoleic acid (CLA) has been reported to have inconsistent effects on bone mass [1-13] and on the differentiation and function of cultured bone cells [3,14-16]. Of these studies, only a few have examined the effects of CLA on bone resorption *in vivo *[5,7,9,11-13] and on osteoclast formation and function *in vitro *[14]. Moreover, none of these studies have examined the effects of the individual bioactive 9*cis*,11*trans *(9,11) and 10*trans*,12*cis *(10,12) CLA isomers on osteoclast formation and activity from cells of human origin.

All of the studies conducted to date on the effects of CLA on bone resorption and osteoclast formation or function have used mixed preparations of CLA, which contain approximately equal amounts of the bioactive 9,11 and 10,12 isomers. Because the individual isomers of CLA could have different effects on bone physiology, it is important to examine the effects of separate preparations of the major biologically active isomers.

The 9,11, but not 10,12, isomer of CLA has been shown to increase mineralized bone nodule formation from human osteoblast-like SaOS-2 cells [15]. These findings suggest that 9,11 CLA may also affect bone resorption based on the knowledge that osteoblasts regulate osteoclast formation by altering the production of receptor activator of NF-κB ligand (RANKL) and osteoprotegerin (OPG) [17], and that CLA modulates RANKL signalling in murine osteoclasts *in vitro *[14]. Osteoclast precursors express receptor activator of NF-κB (RANK), which when activated by the binding of RANKL [18], induces osteoclast differentiation and the expression of osteoclast-specific genes such as those encoding tartrate-resistant acid phosphatase (TRAP), cathepsin K and matrix metalloproteinase-9 (MMP-9) [19]. In contrast, OPG acts as a decoy receptor to inhibit bone resorption by binding to RANKL, which prevents the binding of RANKL to RANK and, therefore, inhibits osteoclast formation and activity. TRAP, cathepsin K and MMP-9 are commonly used as markers of osteoclast formation, and are involved in bone resorption by dissolving mineral (TRAP) [20] and degrading hydroxyapatite (cathepsin K [21] and MMP-9 [22]). Serum concentrations of TRAP have been shown to correlate with the rate of bone resorption *in vivo *[23,24]. *In vitro*, the quantity of TRAP released into cell culture media correlates with bone resorption when osteoclasts are seeded onto bone slices [25].

RANKL belongs to the tumour necrosis factor (TNF) super-family [26,27]. TNFα is a potent pro-inflammatory and bone-resorbing cytokine that binds to the TNF receptor 1 (TNFR1) in osteoclast precursors and augments the stimulatory effects of RANKL on osteoclast differentiation [28,29] thereby inducing bone resorption *in vitro *[30] and *in vivo *[31]. Moreover, when RANKL interacts with RANK, TNFα mRNA expression is upregulated and the TNFα protein is released from osteoclast progenitors to stimulate osteoclast differentiation [29]. Mixed CLA isomers have variable effects on TNFα gene expression [32-35] and inhibit TNFα-induced inflammatory processes [36,37]. In murine RAW264.7 cells, mixed CLA isomers inhibit osteoclast formation and activity by modulating RANKL signalling, as evidenced by a reduction in RANKL-induced TNFα [14], suggesting that CLA may also inhibit TNFα gene expression in human osteoclast precursors.

The objectives of this study were to determine the direct effects of the individual 9,11 and 10,12 isomers on osteoclast formation, activity and osteoclastogenic gene expression from cells of human origin.

## Results

### Effects of CLA on the formation of TRAP^+ ^multinucleated cells from CD14^+ ^monocytes

**Figure 1:**
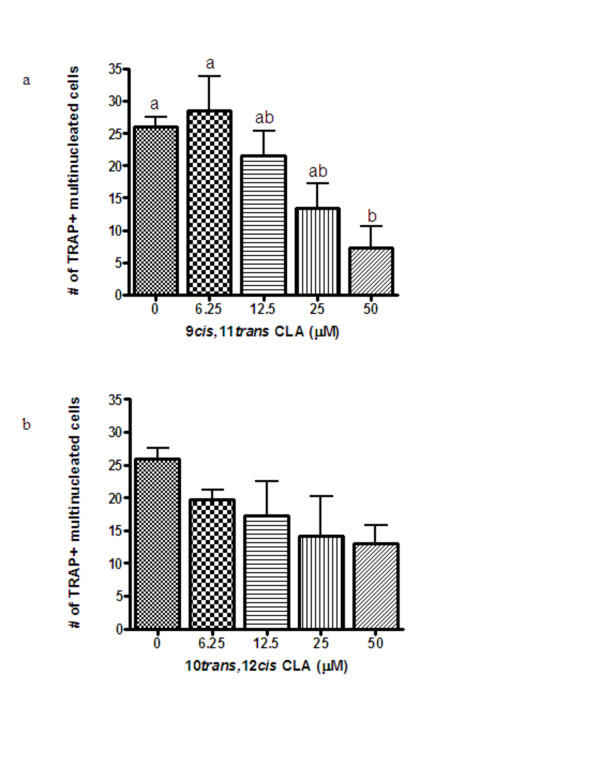
**Effects of increasing concentrations of 9,11 (Figure 1a) and 10,12 (Figure 1b) CLA on osteoclast formation from CD14^+ ^monocytes after 14 days of treatment as determined by the number of TRAP^+ ^multinucleated (≥ 3 nuclei) cells**. Values (n ≥ 3) are expressed as the mean ± SEM of one experiment and the results have been replicated in a second independent experiment. Bars with different letters have significant differences at P < 0.05.

Multinucleated cells were first observed under a phase-contrast microscope (100× magnification) after 10 days of treatment (Day 13). As demonstrated in Figure 1a, 50 μM 9,11 CLA decreased osteoclast formation by ~70%. In contrast, 10,12 CLA had no effect on osteoclast formation (Figure 1b).

### Effects of CLA on TRAP activity in culture media from CD14^+ ^monocytes

**Figure 2:**
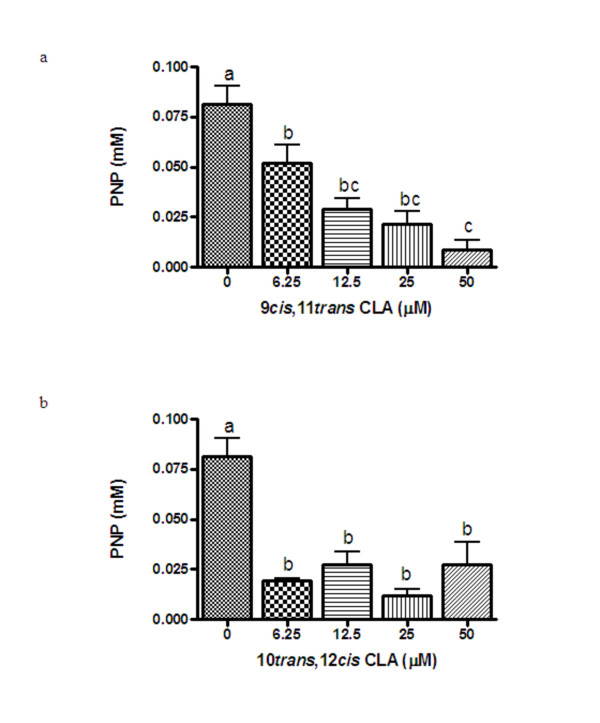
**Effects of increasing concentrations of 9,11 (Figure 2a) and 10,12 (Figure 2b) CLA on TRAP released into the media from CD14^+ ^monocytes on day 14 of treatment**. Values (n = 4) are expressed as the mean ± SEM of one experiment and the results have been replicated in a second independent experiment. Bars with different letters have significant differences at P < 0.05.

TRAP released into the medium was first measurable after 6 days of treatment and increased with duration of treatment. Figure 2 shows the effects of CLA on TRAP activity in the culture media after 14 days of treatment. As seen in Figure 2a, 9,11 CLA inhibited TRAP activity at all concentrations examined in a dose-dependent manner by ~35% (6.25 μM) to 90% (50 μM). The 10,12 isomer of CLA also reduced TRAP activity in the culture media (~67–85%), but there was no dose-response effect at the concentrations tested (Figure 2b).

### Effects of CLA on cathepsin K, RANK, MMP-9 and TNFα gene expression in CD14^+ ^monocytes

The effects of 9,11 and 10,12 CLA on cathepsin K, RANK, MMP-9 and TNFα gene expression were tested after 3 days of treatment. As seen in Table 1, 12.5 to 50 μM of both 9,11 and 10,12 CLA reduced cathepsin K gene expression by ~40–60%. At 6.25–50 μM of 9,11 CLA, RANK expression was reduced by ~60–90%, while 50 μM 10,12 CLA reduced RANK expression by ~70%. Neither 9,11 nor 10,12 CLA affected MMP-9 or TNFα expression.

**Table 1:** Gene expression in CD14^+ ^monocytes following treatment with CLA for 3 days^a^

	Cathepsin K	RANK	MMP-9	TNF-α
9*cis*,11*trans *CLA (μM)				
**0**	1.00^a^	1.00^a^	1.00^a^	1.00^a^
6.25	0.88 ± 0.15^ab^	0.72 ± 0.04^ab^	1.15 ± 0.17^a^	1.08 ± 0.07^a^
12.5	0.59 ± 0.08^bc^	0.50 ± 0.16^b^	0.85 ± 0.16^a^	1.14 ± 0.16^a^
25	0.44 ± 0.03^c^	0.50 ± 0.05^c^	1.00 ± 0.07^a^	0.99 ± 0.17^a^
50	0.43 ± 0.05^c^	0.14 ± 0.05^c^	0.82 ± 0.10^a^	0.91 ± 0.10^a^
				
10*trans*,12*cis *CLA (μM)				
0	1.00^a^	1.00^a^	1.00^abc^	1.00^abc^
6.25	0.80 ± 0.09^a^	0.66 ± 0.07^b^	1.22 ± 0.06^b^	1.19 ± 0.08^abc^
12.5	0.55 ± 0.06^b^	0.60 ± 0.12^bc^	0.88 ± 0.10^c^	1.40 ± 0.19^b^
25	0.53 ± 0.09^b^	0.55 ± 0.09^bc^	0.86 ± 0.07^c^	0.96 ± 0.09^abc^
50	0.47 ± 0.14^b^	0.37 ± 0.02^bc^	0.66 ± 0.08^c^	0.85 ± 0.09^c^

^a^Values represent the mean ± SEM (n ≥ 3), expressed as relative arbitrary units (R.A.U.) for mRNA expression, determined by real-time RT-PCR. Values with different letters represent significant differences within isomer treatment at P < 0.05.

## Discussion

The present study demonstrates that 9,11 CLA, the most abundant isomer found in food products from ruminant animals, inhibits osteoclast formation (TRAP^+ ^multinucleated cells) from human CD14^+ ^monocytes. This effect was accompanied by a decrease in TRAP activity and a reduction in both cathepsin K and RANK gene expression. The 10,12 isomer of CLA appeared to also inhibit osteoclast formation, however, this effect was not significant (p = 0.07). Consistent with a possible inhibitory effect of 10,12 CLA on osteoclast formation, this isomer also reduced osteoclast activity. The results demonstrating no dose-response effect of 10,12 CLA on osteoclast activity, yet a suppression of activity at all concentrations tested, suggests that lower doses of 10,12 CLA may be effective at inhibiting osteoclast activity. The reduction in osteoclast activity following treatment with 10,12 CLA was also accompanied by a reduction in cathepsin K and RANK expression. Our data demonstrate that 9,11 CLA strongly inhibits osteoclast formation from CD14^+ ^monocytes, and suggest that 10,12 CLA may also inhibit osteoclast formation from these cells. These effects appear to be specific to CLA, rather than a non-specific effect of treatment with fatty acids, because linoleic acid did not inhibit osteoclast formation from these cells (data not shown).

To our knowledge, this is the first study to examine the effects of CLA on osteoclast formation and activity from human cells and to examine the effects of the individual 9,11 and 10,12 isomers on osteoclast function. Our results are consistent with the findings from a previous study that showed that mixed CLA isomers reduced osteoclast formation from murine RAW264.7 monocytes [14]. In those cells, 25–100 μM mixed CLA isomers reduced osteoclast formation, as determined by the number of TRAP^+ ^multinucleated cells, in a dose-dependent manner. At 50 μM, the mixed CLA isomers also reduced resorptive pit formation on Osteoclast Activity Assay Substrate plates, while 100 μM mixed CLA isomers reduced TRAP, cathepsin K and MMP-9 gene expression [14]. However, this study used mixed CLA isomers and, therefore, the individual effects of 9,11 and 10,12 CLA were not examined. Our results suggest that the inhibitory effect of mixed CLA isomers on osteoclast formation and activity observed in murine RAW264.7 cells was likely due to the combined effects of both the 9,11 and 10,12 isomers. Moreover, the results from murine RAW264.7 and human CD14^+ ^monocytes suggest that CLA inhibits osteoclast formation in both rodents and humans.

Unlike the experiments in RAW264.7 cells, 100 μM of the individual isomers of CLA appeared to induce apoptosis or were toxic to human CD14^+ ^monocytes. After treatment with 100 μM of either 9,11 or 10,12 CLA the number of adherent CD14^+ ^monocytes was drastically reduced (data not shown). These cells were unable to proliferate and, therefore, the reduction in cell number likely represents an increase in apoptosis or a toxic effect of 100 μM of 9,11 or 10,12 CLA. CD14^+ ^monocytes may be more sensitive to higher concentrations (100 μM) of CLA. In addition, the negative effect of CLA on CD14^+ ^monocytes may be due to higher levels of the individual isomers. The 100 μM solution of mixed CLA isomers represents approximately 40% of 9,11 and 10,12 CLA and, therefore, approximately 40 μM of each individual isomer. This concentration is lower than the maximum concentration (50 μM) of the individual isomers reported in the present study. In addition, RAW264.7 cells are clonal and more robust than primary cells (CD14^+ ^monocytes) and may, therefore, be more resistant to any toxic effects of CLA.

In murine RAW264.7 cells, CLA inhibits osteoclast formation and activity by modulating RANKL signalling, as demonstrated by an inhibition of NF-κB and TNF-α expression. RANKL, which is produced and secreted by osteoblasts, regulates osteoclast differentiation and activity by binding to RANK on the surface of osteoclast precursors. Thus, any effects of CLA on RANK expression may also affect osteoclast formation and activity. The results from the present study demonstrate that both 9,11 and 10,12 CLA inhibit RANK gene expression, supporting the hypothesis that CLA inhibits osteoclast formation by modulating RANKL signalling, and suggest that CLA modulates RANKL signalling by suppressing RANK expression. Although mixed CLA isomers inhibited RANKL-induced TNFα production in murine RAW264.7 cells, individual CLA isomers had no effect on TNFα gene expression in human CD14^+ ^cells. It is possible that CLA inhibits the translation of TNFα mRNA to protein rather than affecting its expression, or that the inhibitory effect of CLA on TNFα protein production is species specific. Although both isomers inhibited cathepsin K gene expression, neither isomer affected MMP-9 expression.

During osteoclast differentiation, MMP-9 is the earliest marker expressed by these cells and is detectable before the precursors become committed to the osteoclast lineage, whereas cathepsin K is predominantly expressed in mature osteoclasts [38]. Thus, the apparent discrepancy between the effects of CLA on cathepsin K and MMP-9 gene expression may be explained by CLA affecting a later stage of differentiation, after the precursors have committed to the osteoclast lineage. In addition, MMP-9 expression is regulated by many growth factors, interleukins and cytokines, including TNFα. As such, the lack of effect of CLA on TNFα expression is consistent with the absence of changes in MMP-9 expression.

Only one study has examined the effect of CLA on bone resorption in humans. This study demonstrated that, compared to placebo (palm/bean oil blend), supplementation with 3 g of CLA per day for 8 weeks had no effect on markers of bone resorption such as serum collagen degradation products, urinary Pyr and dPyr, or serum or urinary calcium levels [13].

Rodent studies demonstrate inconsistent findings regarding the effects of CLA on bone resorption *in vivo *[5,7,9,11,12] and *in vitro *[14]. In young, male mice, a diet containing 0.5% mixed CLA isomers reduced bone resorption after 14 weeks of supplementation compared to a diet containing 0.5% safflower oil [7]. Similarly, in middle aged female mice, a diet supplemented with 9.5% corn oil and 0.5% mixed CLA isomers for 10 weeks decreased serum levels of RANKL and reduced osteoclast function as determined by a reduction in TRAP activity compared to a diet containing 10% corn oil [5]. In adult ovariectomized rats, a diet supplemented with 0.5% or 1% mixed CLA isomers for 9 weeks reduced urinary Pyr crosslinks and dPyr, which are markers of bone resorption, compared to a diet containing 1% soybean oil [11]. In contrast, in young male rats, a diet supplemented with 1% mixed CLA isomers for 8 weeks had no effect on levels of urinary Pyr crosslinks, compared to a diet containing 1% soybean or safflower oil [9]. Similarly, in weanling male rats, a diet containing 6% corn oil and 1% mixed CLA isomers for 8 weeks had no significant effect on bone resorption compared to the control diet containing 7% corn oil [12]. Inconsistencies among animal studies may be due to differences in the species, strain, age and sex of the animals, or to the different doses and durations of CLA treatment [10,13,39-42].

The findings from the present study suggest a beneficial effect of 9,11 CLA on bone mass because osteoclasts degrade bone. When osteoclast mediated bone resorption outweighs osteoblast mediated bone formation, bone loss occurs with each bone remodeling cycle. Compounds that prevent osteoclast formation and activity, such as 9,11 CLA, may prevent bone loss by maintaining the balance between osteoclast and osteoblast activity. This study suggests that CLA inhibits osteoclast formation and activity by modulating RANKL singalling. RANKL is produced and secreted by osteoblasts, and we have previously demonstrated that 9,11 CLA increases osteoblast mediated bone formation *in vitro *[15]. Taken together, the results from these two studies suggest that 9,11 CLA has the potential to improve bone health by maintaining the balance between osteoclast and osteoblast activity during the bone remodeling process, thus preventing bone loss.

## Conclusion

Our findings demonstrate that 9,11 CLA inhibits osteoclast formation and activity from cells of human origin, and suggest that this isomer may prevent bone resorption in humans. The results also suggest that 10,12 CLA inhibits osteoclast formation and activity, but to a lesser degree than 9,11 CLA. The inhibition of RANK expression by both 9,11 and 10,12 CLA is consistent with the hypothesis that CLA inhibits osteoclast formation by modulating RANKL signalling. Although isolated cells are useful for testing directly the effects of purified CLA isomers on osteoclast function, they do not account for differences in CLA metabolism or bone physiology, which are both affected by a number of factors including age, sex, diet and genetic variability. Findings from this study warrant further investigation of the effects of these individual isomers on bone resorption *in vivo*.

## Methods

### Materials

The CD14^+ ^monocytes (2W-400), which are capable of forming osteoclasts *in vitro*, were obtained from Lonza Walkersville (Walkersville, Maryland, USA). The 9,11 and 10,12 (> 98% pure) isomers of CLA were purchased from Matreya (Pleasant Gap, Pennsylvannia, USA). Fetal bovine serum (FBS) was purchased from Cansera (Etobicoke, Ontario, Canada). Antibiotic-antimycotic was purchased from GIBCO (Burlington, Ontario, Canada) and α-MEM and PBS were purchased from the Central Technical Services, University of Toronto. M-CSF and RANKL were obtained from MJS BioLynx Inc. (Brockville, Ontario, Canada). All other chemicals were purchased from Sigma Chemical Co. (St. Louis, Missouri, USA).

### Cell Culture

The CD14^+ ^monocytes obtained from Lonza were isolated from the peripheral blood of screened, healthy donors, by apheresis followed by density centrifugation to remove red blood cells and neutrophils. CD14^+ ^monocytes were isolated using positive immunomagnetic selection directed against the cell surface marker, CD14.

CD14^+ ^monocytes were seeded in basal media (α-MEM containing 10% FBS and 1% antibiotic-antimycotic solution) in 96-well plates at a density of 10^5 ^cells per well. The following day, the medium was replaced with basal media supplemented with 30 ng/ml each of M-CSF and RANKL (differentiation media). On the third day the cells were treated with varying concentrations (6.25, 12.5, 25, 50 and 100 μM) of 9,11 or 10,12 CLA or vehicle (0.1% ethanol and 1 g/L of fatty acid-free BSA). The media were replaced two times per week during the two weeks of treatment.

### CLA Sample Preparation

CLA was added to the medium by first dissolving it in ethanol, which was then added to FBS supplemented with 1 g/L of fatty acid-free bovine serum albumin (BSA) to produce a 10× stock of CLA. The CLA-FBS-BSA solutions were stored at -20°C until needed. These solutions were diluted in serum-free differentiation medium, containing no FBS, prior to each experiment to produce 1× concentrations of CLA and a final FBS concentration of 10%. The final concentration of ethanol and BSA in each well was 0.1% and 1 g/L, respectively.

### Quantification of Osteoclast Formation

Osteoclasts were identified as TRAP^+ ^cells containing 3 or more nuclei. The wells were viewed field by field under a phase-contrast microscope at 100× magnification, and the total numbers of osteoclasts per well were quantified as the sum of each field. For TRAP staining, the cells were fixed with 2.5% gluteraldehyde for 5 minutes, washed two times with PBS that was preheated to 37°C and treated with TRAP stain for 20 minutes at 37°C. TRAP staining was carried out using the protocol described in BD Biosciences Technical Bulleting #445. The TRAP staining solution consisted of 50 mM acetate buffer, 30 mM sodium tartrate, 0.1 mg/ml Naphthol AS-MX phosphate, 0.1% Triton X-100, and 0.3 mg/ml Fast Red Violet LB. After staining, the cells were washed twice with dH_2_O and maintained in dH_2_O.

### Determination of TRAP (Tartrate-Resistant Acid Phosphatase) Activity

TRAP activity was determined in cultured media using an adapted Sigma protocol as described [43]. Briefly, media were added to ELISA plates containing the phosphatase substrate *p*-nitrophenyl phosphate (PNPP) and 40 mM tartrate acid buffer and incubated at 37°C for 30 minutes. The reaction was stopped with the addition of 2N NaOH, and absorbance was measured at 405 nm. TRAP catalyzes the conversion of PNPP to *p*-nitrophenol (PNP), which has a maximal absorbance at 405 nm, and represents TRAP activity in the sample. TRAP activity was calculated from a standard curve obtained from PNP standards.

### RNA Isolation and Real-Time, One-Step RT-PCR

After 3 days of treatment, the cells were washed twice with PBS and lysed in 0.5 ml of nucleic acid purification solution (Applied Biosystems). Total RNA was isolated using the 6100 Nucleic Acid PrepStation (Applied Biosystems). A one-step reaction was performed in an ABI Prism^® ^7000 Sequence Detection System (Applied Biosystems) to reverse transcribe the mRNA into cDNA, which was then amplified using the QuantiTect Multiplex RT-PCR Kit (Qiagen) and TaqMan^® ^Gene Expression Assays (Applied Biosystems). All reactions were performed in 96-well plates with a final volume of 25 μl per well. Cycling conditions were 20 minutes at 50°C followed by 15 minutes at 95°C to activate the HotStar Taq DNA Polymerase, and 50 cycles of 45 seconds at 94°C, and 45 seconds at 60°C. The TaqMan^® ^Gene Expression Assays used for cathepsin K, RANK, MMP-9 and TNFα were Hs01080388_m1, Hs00187189_m1, Hs00957555_m1 and Hs00174128_m1, respectively. The target genes were co-amplified with VIC-labelled β-2-microglobulin (Applied Biosystems, #4326319E) as an internal control. Data were obtained as threshold cycle (C_T_) values, which represent the cycle at which the first significant increase in fluorescence is detected, and corresponds to the amount of starting template in the sample. The difference in C_T _values (ΔC_T_) between the internal control (VIC-labeled β-2-microglobulin) and target gene of interest (FAM-labelled) was calculated to determine the relative change in C_T _values between samples. The average ΔC_T _of control samples was subtracted from the ΔC_T _of treatment samples to derive a ΔΔC_T_value, which represents the change in mRNA expression between treatments relative to controls. Relative mRNA levels were calculated as 2^-ΔΔCT ^and expressed as fold change relative to control samples that produce a 2^-ΔΔCT ^value of 1.

### Statistical Analyses

Results are expressed as mean ± SEM with at least 3 replicates in each group. Differences were analyzed using a one-way ANOVA followed by Tukey's test for multiple comparisons. P values < 0.05 were considered significant. All data were analyzed using GraphPad Prism Software, Version 4.0.

## Competing interests

The authors declare that they have no competing interests.

## Authors' contributions

IP helped conceive the study, participated in the design of this study, carried out the experiments, performed statistical analyses and drafted the manuscript. AEL helped conceive the study, obtained funding, participated in the design and coordination of the study and helped draft the manuscript. All authors read and approved the final manuscript.
